# Exploring strain diversity of dominant human skin bacterial species using single-cell genome sequencing

**DOI:** 10.3389/fmicb.2022.955404

**Published:** 2022-08-05

**Authors:** Keigo Ide, Tatsuya Saeki, Koji Arikawa, Takuya Yoda, Taruho Endoh, Ayumi Matsuhashi, Haruko Takeyama, Masahito Hosokawa

**Affiliations:** ^1^Department of Life Science and Medical Bioscience, Waseda University, Tokyo, Japan; ^2^Computational Bio Big-Data Open Innovation Laboratory, National Institute of Advanced Industrial Science and Technology, Tokyo, Japan; ^3^bitBiome, Inc., Tokyo, Japan; ^4^Research Organization for Nano and Life Innovation, Waseda University, Tokyo, Japan; ^5^Institute for Advanced Research of Biosystem Dynamics, Waseda Research Institute for Science and Engineering, Tokyo, Japan

**Keywords:** skin, microbiome, single-cell genomics, metagenomics, genome

## Abstract

To understand the role of the skin commensal bacterial community in skin health and the spread of pathogens, it is crucial to identify genetic differences in the bacterial strains corresponding to human individuals. A culture-independent genomics approach is an effective tool for obtaining massive high-quality bacterial genomes. Here we present a single-cell genome sequencing to obtain comprehensive whole-genome sequences of uncultured skin bacteria from skin swabs. We recovered 281 high-quality (HQ) and 244 medium-quality single-amplified genomes (SAGs) of multiple skin bacterial species from eight individuals, including cohabiting group. Single-cell sequencing outperformed in the genome recovery from the same skin swabs, showing 10-fold non-redundant strain genomes compared to the shotgun metagenomic sequencing and binning approach. We then focused on the abundant skin bacteria and identified intra-species diversity, especially in 47 *Moraxella osloensis* derived HQ SAGs, characterizing the strain-level heterogeneity at mobile genetic element profiles, including plasmids and prophages. Even between the cohabiting individual hosts, they have unique skin bacterial strains in the same species, which shows microdiversity in each host. Genetic and functional differences between skin bacterial strains are predictive of *in vivo* competition to adapt bacterial genome to utilize the sparse nutrients available on the skin or produce molecules that inhibit the colonization of other microbes or alter their behavior. Thus, single-cell sequencing provides a large number of genomes of higher resolution and quality than conventional metagenomic analysis and helps explore the skin commensal bacteria at the strain level, linking taxonomic and functional information.

## Introduction

The skin is the largest human organ and is constantly exposed to indigenous microbiota. Skin commensal bacteria play an important role in skin health and deter the spread of pathogens (Iwase et al., 2010; Zipperer et al., 2016; Williams et al., 2019). Studies of skin commensal bacteria such as *Cutibacterium acnes* and *Staphylococcus epidermidis* have shown that their specific strains are associated with skin disease states in their human hosts (Conlan et al., 2012; Tomida et al., 2013). In addition, skin commensal bacteria respond to the surrounding skin bacterial community, switching their function from virulence to commensalism (Ramsey et al., 2016). Toward understanding the function of the skin commensal bacterial community, it is crucial to focus on the microbial compositions and identify genetic and functional differences in the bacterial strains corresponding to human individuals or body sites and how these differences correlate to skin health.

While previous studies on skin bacteria have taken a culture-dependent approach, a culture-independent approach based on metagenomic analysis has recently been used. The approach that combined existing bacterial culture methods with shotgun metagenomic analysis constructed the Skin Microbial Genome Collection (SMGC), a database of human skin bacteria genomes (Saheb Kashaf et al., 2022). This database reports that bacteria belonging to *Cutibacterium*, *Corynebacterium*, *Staphylococcus*, *Lawsonella*, and *Candidatus* Pellibacterium are the major bacterial species on the skin. With large-scale shotgun metagenomic analyses available, investigations on the characterization of indigenous skin bacteria by race have also been conducted. In particular, a study showed a greater abundance of *Moraxella osloensis* in Chinese skin than in Westerners, suggesting that the microbial trophic chains of *M. osloensis* affect skin health (Li et al., 2021). In addition, a meta-analysis of public metagenomic data has acquired skin bacterial metagenome-assembled genomes (MAGs) across age, geography, and lifestyle (Pasolli et al., 2019). Another study has shown that deep metagenomic long-read sequencing using PacBio SMRT sequencing can identify previously unknown *Corynebacterium simulans* and its companion bacteriophages from high-quality closed MAGs (Tsai et al., 2016). Thus, a culture-independent approach using state-of-the-art sequencing technology is essential to reveal the unexplored ecology of skin commensal bacteria and support functional understanding *via* analysis of high-quality bacterial genomes.

Shotgun metagenomics is able to provide comprehensive genetic information on microbial communities, but linking taxonomic and functional information for individual bacterial strain genomes remains a challenge (Scholz et al., 2016; Van Rossum et al., 2020). In metagenomics, the construction of MAG involves assembling fragmented genome sequences into contigs, then binning the contigs into lineages. However, metagenomic binning produces incomplete bins as well as multi-species composite bins that aggregate sequences originating from multiple distinct species or strains (Shaiber and Eren, 2019). We have found that the *Staphylococcus* MAGs from the human skin microbiota show high completeness (83–94%) while showing more than a twofold difference in genome sizes between MAGs generated from different metagenome binning tools (Arikawa et al., 2021), which either suggests a substantial lack of accessory genes or an excess of sequences from contamination with other closely related *Staphylococcus* species. The binning tools rely on common nucleotide compositional characteristics such as tetranucleotide frequency, GC content, or sequence coverage (Alneberg et al., 2014; Kang et al., 2019). However, this genome reconstruction scheme makes the relationships between bacterial host and mobile genetic elements (MGEs), such as plasmids and phages, difficult to delineate when the community has multiple strains within the same species.

Single-cell genomics is an alternative approach to culture-independent sequencing of microbial genomes (Woyke et al., 2017). In contrast to metagenomics, single-cell genomics does not require microbial population clonality but instead recovers genome sequences from individual cells. We have developed microfluidic technology-aided single bacterial genome sequencing, called SAG-gel, to obtain a large number of single-amplified genomes (SAGs) (Chijiiwa et al., 2020; Arikawa et al., 2021; Hosokawa et al., 2022). SAG-gel uses microfluidic-generated gel capsules that enable single-cell encapsulation and sequential enzymatic reactions for cell lysis and genome amplification of single cells in a parallel manner. We have demonstrated its applicability for obtaining strain-resolved genomes from mice and human microbiome and elucidating their metabolic function and host-plasmid association in the microbial community.

In this study, we applied SAG-gel to obtain massive single-amplified genomes (SAGs) of multiple human skin bacterial species. To demonstrate the advantages of our single-cell approach for obtaining strain-resolved bacterial genomes from human skin, we compared the number, quality, and diversity of the bacterial genomes obtained from healthy facial skins to the conventional metagenomic binning approach. We also validated the potential of single-cell genome sequencing for revealing the intra-species microdiversity of human skin microbiota by linking MGEs with host bacterial genomes.

## Materials and methods

### Sample collection

Skin swab samples were collected from healthy volunteers without skin disease. After waking up in the morning without washing their face, participants swabbed their facial skin surface using sterile cotton applicators (Nissui Pharmaceutical Co., Ltd.) pre-moistened with Dulbecco’s phosphate-buffered saline (DPBS) by the participants. The swabs were stored in DPBS at room temperature for 2 days maximum, prior to DNA extraction and single-cell genome amplification.

### Metagenomic sequencing

The DNeasy Power Soil Pro Kit (QIAGEN) was used for total DNA extraction from skin swab samples. Metagenomic sequencing libraries were constructed from extracted DNA samples with 10-μL (1/5 volume) reactions of the QIAseq FX DNA Library Kit. Each metagenomic sequencing library was sequenced using the Illumina HiSeq 2 × 150 bp configuration (Macrogen).

Metagenomic reads (MRs) were processed in the ATLAS pipeline v2.6a2 (Kieser et al., 2020), using fastp v0.20.1 (Chen et al., 2018) for quality control and Maxbin v2.2.7 (Wu et al., 2016) and Metabat2 v2.15 (Kang et al., 2019) for binning as default parameters, then refined in DAS_tool v1.1.4 (Sieber et al., 2018). For each sample, dRep v.2.2.3 (Olm et al., 2017) was run to obtain the highest quality genome from the genome with 99% average nucleotide identity (ANI). The relative bacterial composition of the samples was obtained by running Kraken2 v2.1.2 (Wood et al., 2019) against the GTDB release95 database constructed by struo2 v2.2.2 (Youngblut and Ley, 2021).

### Single-cell genome sequencing with SAG-gel

According to our previous reports, single-cell genome sequencing was performed with single-cell whole genome amplification (WGA) using the SAG-gel platform (Chijiiwa et al., 2020). For sequencing analysis, sequencing SAG libraries were prepared from second-round MDA products using the QIAseq FX DNA Library Kit (QIAGEN). Ligation adaptors were modified to TruSeq–Compatible Full-length Adapters UDI (Integrated DNA Technologies). Each SAG library was sequenced using the Illumina HiSeq 2 × 150 bp configuration (Macrogen).

### Pre-processing and assembly of single-cell genomic reads

Single-cell genomic reads (SRs) were individually processed to eliminate LQ reads by using fastp v0.20.1 (Chen et al., 2018) with default options or BBDuk v38.79 (Bushnell et al., 2017) (options: qtrim = r, trimq = 10, minlength = 40, maxns = 1, minavgquality = 15, ktrim = r, ref = adapters k = 23 mink = 11 hdist = 1 tpe tbo). Human genome contamination was removed from the sequence reads by mapping to the reference human genome with BBMap v38.79 [options: quickmatch fast untrim minid = 0.95, maxindel = 3, bwr = 0.16, bw = 12, minhits = 2, path = human_masked_index (*), qtrim = rl, trimq = 10]. Short reads were assembled *de novo* using SPAdes v3.14.0 (options for SAG: −sc -careful -disable-rr -disable-gzip-output −t 4 −m 32), and contigs < 1,000 bp were excluded from subsequent analyses (Bankevich et al., 2012).

### Grouping same strain single-amplified genomes into CoSAG

SAGs with completeness 20% and contamination of < 10% were selected with CheckM v1.1.2 (Parks et al., 2015). ANI was calculated for selected SAGs using enveomics (Rodriguez-R and Konstantinidis, 2016). The homology of common single-copy marker genes obtained using CheckM taxonomy workflow (option: −nt –tab_table −t 16 domain Bacteria) was calculated by blastn v2.9.0 + with the default option. SAGs with ANI > 99%, single-copy marker gene homology > 99%, and tetra-nucleotide frequencies correlation > 90% were identified in the same strain group.

SRs from one SAG were mapped to other SAGs in the same group using MINIMAP2 v2.17 (options: −ax sr) (Li, 2018). According to the ccSAG procedure (Kogawa et al., 2018), partially aligned potential chimeras were split into aligned and unaligned fragments. The short fragments (< 20 bp) were discarded. Clean and chimera-removed reads were obtained using cycles of cross-reference mapping and chimera splitting for each sample in the same group. Quality-controlled reads from the same group were co-assembled *de novo* as CoSAG using SPAdes (options: –sc –careful –disable-rr –disable-gzip-output −t 4 −m 32).

### Gene prediction, taxonomy identification, and plasmid detection

CDS, rRNAs, and tRNAs were extracted from all SAGs or MAGs through Prokka 1.14.6 (Seemann, 2014) (option: –rawproduct –mincontiglen 200). Thereafter, 16S and 23S rRNA genes were detected with lengths ≥ 700 and 1,100 bp, respectively. Taxonomy identification was performed using GTDB-Tk 1.3.0 (Chaumeil et al., 2019) with the default option, using the Release95 database.

### Functional annotation, orthogroups analysis, and phylogenetic analysis

Eggnog and InterProScan v5.40-77.0 (Jones et al., 2014) were performed on the predicted genes for functional annotation. Orthogroups analysis using Orthofinder v2.3.0 (Emms and Kelly, 2019) was performed for *M. osloensis* and *S. epidermidis*. PlasForest v1.3 (Pradier et al., 2021) was used to detect plasmids from genome sequences. ggtree v3.0.4 (Yu et al., 2017), ggtreeExtra v1.2.3 (Xu et al., 2021), and aplot v0.1.3 were used for phylogenetic trees and visualization of the results. gggenomes v0.9.5.9000 was used to visualize the synteny regions among genomes.

## Results

### Overview of skin single-amplified genome collection

**FIGURE 1:**
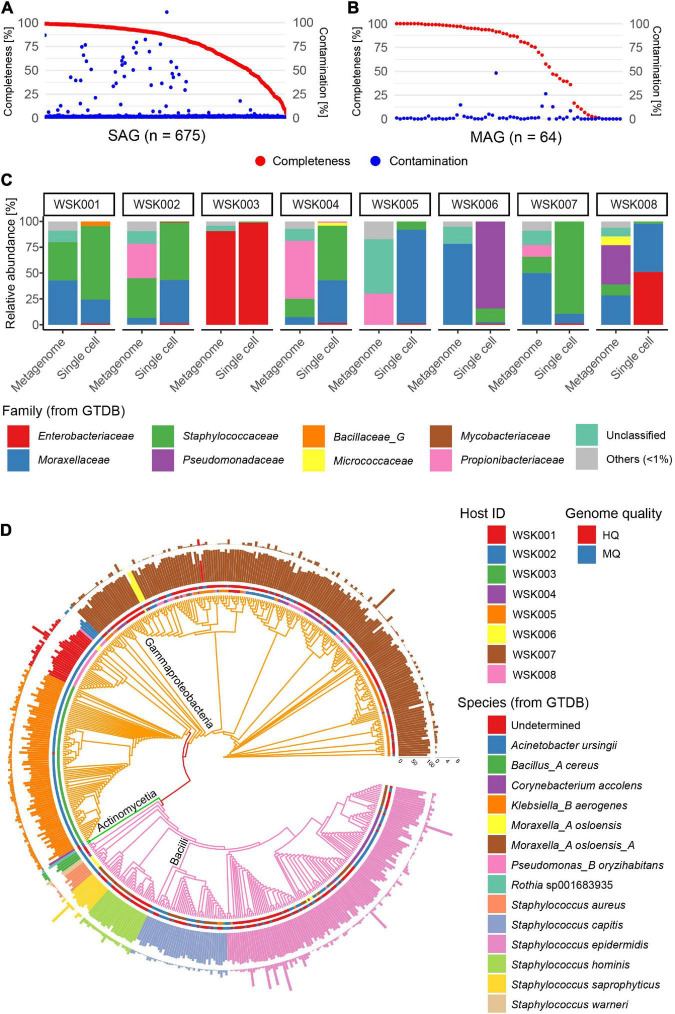
Single-amplified genomes (SAGs) obtained from human skin bacteria. **(A,B)** Completeness and contamination statistics for **(A)** 675 SAGs and **(B)** 64 metagenome-assembled genomes (MAGs) that were obtained from 8 adult subjects, including three cohabitation groups (group1: WSK001 and WSK002, group2: WSK003 and WSK004, group3: WSK007 and WSK008). **(C)** Relative abundance profiles of skin bacteria in 8 individuals. Box plots indicate the phylogenetic composition calculated based on SAG numbers or metagenomic reads. **(D)** Taxonomic annotation of high-quality (Completeness > 90%, Contamination < 5%) and medium-quality (Completeness ≥ 50%, Contamination < 10%) SAGs. Inner circle: host; outer circle: SAG quality; inner bar: completeness; outer bar: contamination rate. Bars are colored by species. Tree edges are colored by family.

Skin swab samples were collected from eight participants without skin disease. Participant characteristics are shown in Supplementary Table 1 and included four Japanese adult males and four females each (aged 29–46 years), including three cohabiting groups. A total of 675 SAGs (71–89 SAGs per subject) and 64 MAGs (3–18 MAGs per subject) were obtained from 8 samples [mean completeness: 77.6% (SAG) and 67.2% (MAG), mean contamination rate: 3.7% (SAG) and 2.7% (MAG)] (Figures 1A,B). Of these, 628 valid SAGs were obtained, excluding SAGs with > 10% contamination, which consisted of 281 high-quality (HQ, > 90% completeness, < 5% contamination) SAGs, 244 medium-quality (MQ, ≥ 50% completeness, < 10% contamination) SAGs, and 103 Low-quality (LQ, < 50% completeness, < 10% contamination) SAGs (Supplementary Table 2).

The bacterial phylogenetic trends of the obtained SAGs differed between human hosts (Figures 1C,D), and the bacterial compositions based on SAGs and shotgun metagenomics showed that some bacteria were common in the same host. However, their compositional proportions were not consistent. An average of 6.0% (0.5–26.5%) of the acquired raw sequence reads in shotgun metagenomics were derived from the contaminated human genome, while 0.02% (0.00–10.8%) of the raw sequence reads in single-cell genomics were derived from the human genome.

**FIGURE 2:**
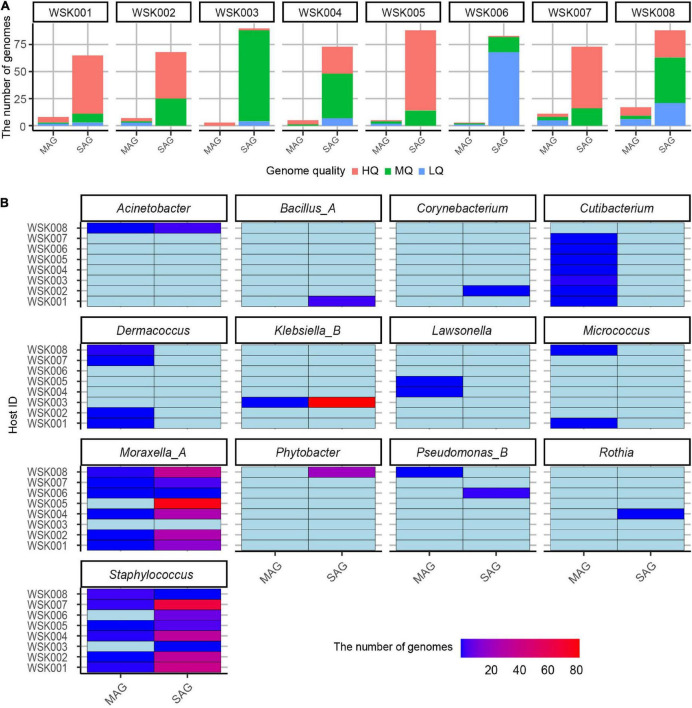
Non-redundant bacterial genomes obtained from human skin bacteria. **(A)** The number of non-redundant single-amplified genomes (SAGs) and metagenome-assembled genomes (MAGs) for eight human hosts. Bars were colored by the genome quality (high-quality: HQ, medium-quality: MQ, low-quality: LQ, and others (high contamination rate: > 10%). **(B)** Genus-level classification of medium-quality SAG and MAGs for each host. The genome abundances were shown on the color scale.

Single-cell genomics yielded 10-fold more non-redundant HQ and MQ genomes than shotgun metagenomics (Figure 2A). Further, multiple non-redundant SAGs from *Moraxella_A osloensis_A* (197 SAG) and *Staphylococcus* spp. (especially *Staphylococcus epidermidis* 115 SAG), *Klebsiella_B aerogenes* (84 SAG) were recovered. These SAGs showed that genomes of multiple unique strain genomes were obtained in the genera *Staphylococcus* and *Moraxella* (Figure 2B). On the other hand, since all MAGs were species-representative genomes, no more than two bacterial strain MAGs of the same species were recovered from the same host.

### Obtaining strain-resolved HQ single-amplified genomes of *Moraxella osloensis* and *Staphylococcus epidermidis*

**FIGURE 3:**
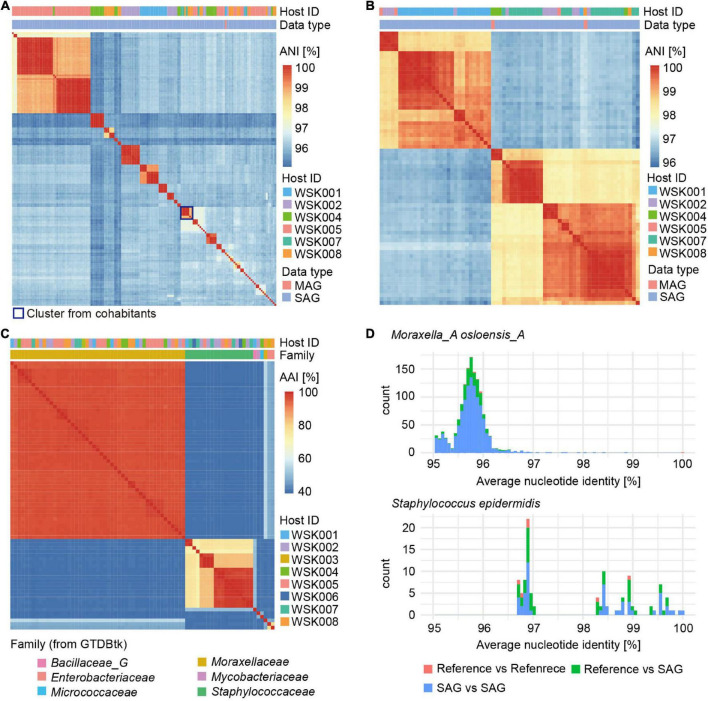
Obtaining strain-resolved genomes for *Moraxella osloensis* and *Staphylococcus epidermidis*. **(A,B)** Clustering of single-amplified genomes (SAGs) and metagenome-assembled genomes (MAGs) for **(A)** *M. osloensis* and **(B)** *S. epidermidis* based on the average nucleotide identity (ANI). Based on the SAG clustering (ANI > 99%, TNF > 90%, single-copy marker gene > 99.9%), composite SAGs (CoSAGs) representing each bacterial strain were constructed for each host. **(C)** Clustering of high-quality (HQ) SAGs for obtaining strain-resolved genomes, including CoSAG, for *M. osloensis* and *Staphylococcus* spp., and other skin bacteria species based on average amino acid identity (AAI). **(D)** Distribution of pairwise ANIs for strain-resolved HQ SAGs of *M. osloensis* and *S. epidermidis* with known reference genomes. In panels **(A–C)**, HQ SAGs (Completeness > 90%, Contamination < 5%) are shown.

Clustering of abundant *Moraxella* and *Staphylococcus* SAGs, based on average nucleotide identity, was used to evaluate groups of bacterial strains (Figures 3A,B). For *Moraxella osloensis*, three or more bacterial strain SAGs were obtained from six individual hosts (maximum 22 SAGs/host, HQ: 155 SAGs, MQ: 47 SAGs). In contrast, only one *M. osloensis* strain MAG was obtained from each of the five hosts (Figure 3A). For *Staphylococcus*, one MAG of *Staphylococcus epidermidis* was obtained from each of the four hosts, while no MAGs were obtained from the other hosts. In contrast, for SAGs, at least one *S. epidermidis* SAG was obtained from 7 hosts, and 4 of the seven hosts had multiple SAGs (maximum 5 SAGs/host, HQ: 70 SAGs, MQ: 52 SAGs) (Figure 3B). Almost all *M. osloensis* and *S. epidermidis* strains were found to be independent per host (Figures 3A,B), but a SAG cluster of *M. osloensis* with extremely high ANI (99.96%) was found in the cohabiting couple WSK007 and WSK008 subjects, which was presumed to be the same strain (Figure 3A). Based on the clustering of these SAGs (ANI > 99%, TNF > 90%, single-copy marker gene > 99.9%), composite SAGs (CoSAGs) representing each bacterial strain were constructed for each host. CoSAGs were constructed for *Moraxellaceae* [31 CoSAGs (28 HQ)], *Staphylococcaceae* [19 CoSAGs (16 HQ)], *Enterobacteriaceae* [2 CoSAGs (2 HQ)], *Bacillaceae_G* [1 CoSAG (1 HQ)], and *Pseudomonadaceae* (1 CoSAG), respectively (Figure 3C and Supplementary Table 3).

Intraspecies ANIs of strain-resolved HQ SAGs in *M. osloensis* or *S. epidermidis* were determined by comparing them to known reference genomes of isolated strains [*M. osloensis*: KMC41 (GCA_002355615.1), KSH (GCF_002752795), TT16 (GCF_002786455), YHS (GCF_002752755), *S. epidermidis*: ATCC 12228 (GCF_000007645), W23144 (GCF_000160235), BVS058A4 (GCF_000314715), CIM40 (GCF_000417985)]. *M. osloensis* SAGs had ANIs ranging from 94.94 to 98.84% with a peak of nearly 96%, suggesting high intra-species genomic diversity (Figure 3D). On the other hand, *S. epidermidis* SAGs had ANIs ranging from 96.70 to 99.99% peaks distributed over 97%, consistent with a previously reported ANI distribution (Chaudhry and Patil, 2016). ANIs between SAGs and reference genomes showed reasonable values (*M. osloensis*: 94.96–96.50% and *S. epidermidis*: 96.73–99.70%), compared to ANIs between references (*M. osloensis*: 95.61–99.99% and *S. epidermidis*: 96.73–98.95%) and no obvious differences were observed in their genome size, GC%, and the number of CDS (Figure 3D and Supplementary Figure 1). One of the *S. epidermidis* MAGs showed an excess genome size of more than 200 kbp and high GC% to the known complete genomes and our SAG, suggesting inaccuracies in contig classification in metagenomic binning (Supplementary Figure 1).

### Pangenome analysis of *M. osloensis* or *Staphylococcus* spp. and annotating plasmids

**FIGURE 4:**
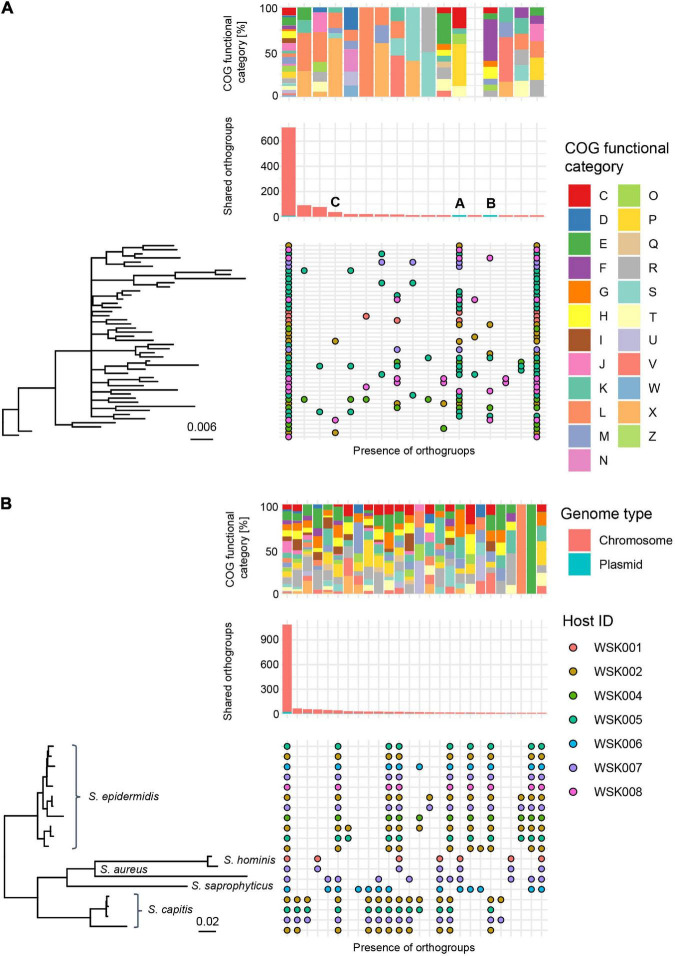
Pangenome analysis of HQ SAGs in *Moraxella osloensis* and *Staphylococcus* spp. **(A,B)** Profiles of orthogroups (OGs) in **(A)** 47 *Moraxella osloensis* and **(B)** 19 *Staphylococcus* spp. (*S. epidermidis*, *S. aureus*, *S. capitis*, *S. hominis*, and *S. saprophyticus*). The upper box shows the composition of the COG category in each gene occurrence pattern. The middle bar plot shows the number of OGs with plasmid prediction. The bottom chart shows the presence/absence of OGs that constitute the pangenome of analyzed HQ SAGs sorted with the phylogenetic tree based on the OGs. Occurrence patterns are shown in which more than 10 OGs were present. No occurrence patterns are shown where OGs are missing in only one HQ SAG of all strains.

Multiple HQ SAGs (Completeness > 90% and Contamination < 5%) obtained from *M. osloensis* and *Staphylococcus* spp. (including *S. aureus*, *S. capitis*, *S. hominis*, *S. saprophyticus* as well as *S. epidermidis*) were subjected to pangenome analysis. In 47 HQ SAGs of *M. osloensis*, 701 (17.4%) of the total 4,038 Orthogroups (OGs) were common to all SAGs. Other OGs were scattered among the strains; many were classified in the X (Mobilome: prophages, transposons) and L (replication, recombination, and repair) categories. Accessory gene occurrence patterns suggest that a certain number of OGs are MGEs that have been propagated to the host genome *via* phage or other means (Figure 4A). In 19 HQ SAGs of *Staphylococcus* spp., 1,089 (33.8%) of the total 3,221 OGs were common to all SAGs (Figure 4B). The OG of *Staphylococcus* spp. is conserved, corresponding to the species clade, showing homogeneous intraspecific genetic diversity compared to *M. osloensis*.

**FIGURE 5:**
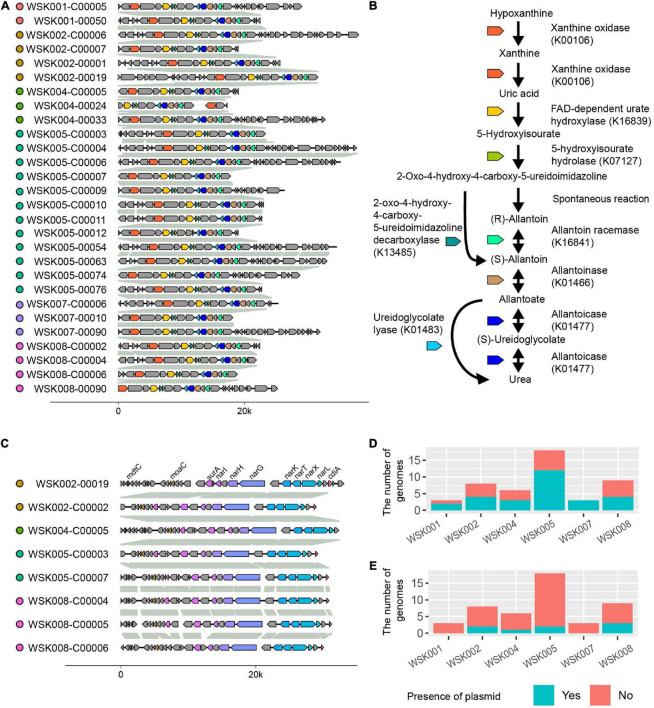
Plasmids found in *Moraxella osloensis* high-quality single-amplified genomes (HQ SAGs). **(A)** Synteny analysis of contigs predicted as plasmid sequence shown in Figures 4A-A. **(B)** Predicted purine metabolic pathway found in the plasmid **(A)**. The pathway degrades xanthine to allantoin as an intermediate metabolite and urea as an end product. **(C)** Synteny analysis of contigs predicted as plasmid sequence shown in Figures 4A-B. The complete nitrate reductase operon conserving nar-related genes is observed. **(D,E)** The numbers of CoSAGs harbor the genes shown in panels **(A,C)** in the plasmid.

As arrows (A and B) indicate in Figure 4A, two gene occurrence patterns were present in *M. osloensis* plasmids. The degradation pathway operon from xanthine to urea, a purine metabolic pathway, was conserved in the gene occurrence A presented in 28 (60.0%) *M. osloensis* HQ SAGs (Figure 5A). This pathway included allantoin as an intermediate metabolite and urea as an end product (Figure 5B). On the other hand, the complete nitrate reductase operon was conserved in the gene occurrence B, a putative plasmid from 8 (17.0%) *M. osloensis* HQ SAGs; specifically, nar-related genes (narK, narL, narK, and others) are conserved in the nitrogen reduction-related gene operon (Figure 5C). A search of the genomes of publicly available *M. osloensis* isolates for these genes found in patterns A and B revealed that they were annotated as plasmids in five isolates each [A: KMC41 (GCA_002355615.1), TT16 (GCA_002786455.2), YHS (GCA_002752755.2), FDAARGOS_1202 (GCA_016888885.1), CCUG 350 (GCA_001553955.1), and B: KMC41 (GCA_002355615.1), TT16 (GCA_002786455.2), YHS (GCA_002752755.2), KSH (GCA_002752795.2), NP7 (GCA_002753715.2)]. This agreement suggested that the plasmid annotation of *M. osloensis* HQ SAG was accurate. Among the acquired *M. osloensis* HQ SAGs, at least one strain for each host possessed the plasmid with the xanthine degradation pathway (A) (Figure 5D). Interestingly, all HQ SAGs of *M. osloensis* derived from WSK007 possessed this plasmid. In contrast, *M. osloensis* from all hosts, except WSK001 and WSK007, possessed the plasmid coding for the nitrogen reduction gene operon (B).

### Identification of prophage in *Moraxella osloensis* genome

**FIGURE 6:**
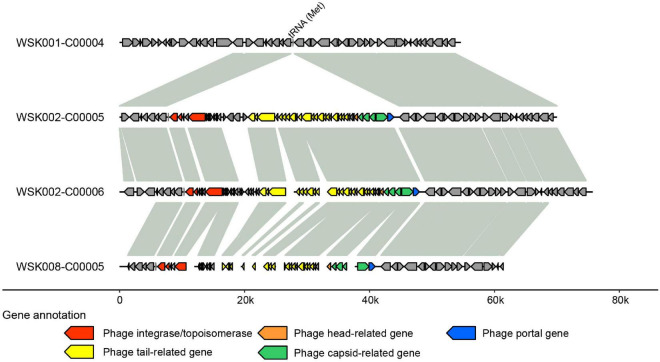
Predicted prophage sequences in *Moraxella osloensis* high-quality single-amplified genomes (HQ SAGs) obtained from WSK002 and WSK008. The genes encoded here are shown in the gene occurrence pattern C in Figure 4A. The genome synteny was compared with the HQ SAG of WSK001.

Thereafter, to understand the commonality of MGE among bacterial strains suggested in Figure 4, we searched for prophages in the genome by synteny comparison of HQ SAGs of *M. osloensis* (Figure 6), leading to the identification of a pair of different strains with a common prophage insertion in the same host (WSK002). A CoSAG with the same prophage insertion was also identified from a different host (WSK008) (the gene occurrence pattern C in Figure 4A). Some of the genes in this region were presumed to be phage-related genes, including phage-tail, phage-head, phage-capsid, and phage-integrase. When the phage inserts its genome into the host genome, one of the insertion mechanisms uses integrase to target the attB region of the tRNA genes (Williams, 2002). In this study, Methionine tRNA was identified in the insertion locus of the prophage, suggesting that the prophage was inserted into the host genome by tRNA-mediated insertion (Figure 6).

## Discussion

This study is the first application of single-cell genomics to acquire massive skin commensal bacterial genomes to the best of our knowledge. Our results indicate that single-cell sequencing can recover more genomes from the skin bacterial community at once than genome sequencing of cultured strains or metagenome sequencing of uncultured bacteria and can evaluate intra-species diversity of skin bacteria, especially in abundant SAGs of *Staphylococcus* and *Moraxella*. No clear relationship was observed between the subject background, and the bacterial species of SAG or MAG obtained because this study included subjects of similar age groups who did not have skin diseases and were not using antibiotics. The individual hosts showed multiple independent *M. osloensis* or *S. epidermidis* strains. It has been reported that *S. epidermidis* has multiple lineages within individuals, and the essential determinant of microbial diversity is the body site for sample collection. Our results obtained multiple strain genomes from individuals were consistent with this finding (Zhou et al., 2020). On the other hand, in one of three cohabiting couples, *M. osloensis*, considered the same strain, was collected from both hosts. This result suggests that the same skin bacterial strains may be shared between individuals who share a living environment and have frequent contact with each other.

It has been reported that shotgun metagenomics showed that *M. osloensis* is a specific species for Chinese skin and skin types with high *M. osloensis* abundance were more common in the elderly individuals (Li et al., 2021). However, previous studies have not focused on the functional genes or MGEs of *M. osloensis*. The metabolic pathway operons from xanthine to allantoin and urea in *M. osloensis* plasmid were not found in other major skin bacteria such as *S. epidermidis*, *S. aureus*, *C. acnes*, or other *Moraxella* spp. This gene specificity suggests that *M. osloensis* has acquired these genes in a species-specific manner by employing plasmids and potentially has unique functional features in the skin microbiome.

Regarding the genes on the plasmids; allantoin, an intermediate product of the metabolic pathway encoded in the *M. osloensis* plasmid, is a substance of interest specifically for its antioxidant and anti-inflammatory properties, antibacterial effects, and wound healing in the skin (Paller et al., 2017). In addition, the end product, urea, is considered a natural moisturizing factor and is important for skin hydration (Piquero-Casals et al., 2021). The nar operon encodes a family of nitrogen reductases that reduce nitrate in the bacterial membrane. Although there are only a few reports on nitrogen reductases in *Moraxella*, it is known that nitrate-reducing bacteria are abundant in skin bacterial populations (Wang et al., 2011; Bewick et al., 2019). *Staphylococcus* species also evolved to be tolerant of high salinity in sweat as a survival strategy on the skin (Byrd et al., 2018). The gene cluster encoded in the *M. osloensis* plasmid, whose existence was revealed by the SAG in this study, may be responsible for carrying the function that adapts *M. osloensis* to the skin environment. As a survival strategy on the skin, similar to *Staphylococcus* spp., *M. osloensis* may maintain high intraspecific diversity in its genome to expand its niche in the skin with propagating plasmids carrying genes involved in nitrate reduction.

Phages of skin bacteria have recently received attention as a potential means of treating various bacterial infections. Overuse of antibiotics is presumed to contribute to the selection of antimicrobial-resistant organisms and subsequent collateral damage to commensal microbes (Jo et al., 2021). In contrast, phage therapy can effectively treat skin bacterial infections while avoiding conventional antibiotics (Shimamori et al., 2020). Although accumulating skin phage information is required to enable practical phage application, skin swabbing cannot acquire sufficient phages (Duplessis et al., 2021). Thus, understanding the ecology of skin phages relies on the metavirome approach (van Zyl et al., 2018). However, metagenomic (metavirome) analysis cannot efficiently predict the host of phages and often misses the presence of prophages inserted into the host bacterial genome. The acquisition of a broad range of strain genomes is crucial to elucidate the relationship between phage and the bacterial strain host. Our study demonstrates that strain-resolved SAG comparison of skin bacteria is suitable for detecting the prophage that exists heterogeneously in the same species genome and within the same host. Because previous research reported no obvious prophage insertion in the complete genome of *M. osloensis* strain KMC41 (Goto et al., 2016), this study is the first to demonstrate phage infection in the *M. osloensis* genome, and it goes on to further suggest that propagation within the species leads to genomic diversity in *M. osloensis*. Our method solves the problem of conventional metavirome approaches because it can capture genes of phage that infect bacteria from high-quality host bacterial genomes.

As shown in our comparison of skin bacterial SAGs and MAGs, single-cell genomics allows us to obtain a large number of HQ genomes with higher resolution than conventional metagenomic binning from skin swab samples. As experimental steps to genome acquisition, single-cell genomics with SAG-gel requires a microfluidic operation, gel capsule isolation into the well plate, and library preparation for index single-cell sequencing. These processes require one to 2 days longer operation time than metagenomic sequencing, consisting of simple DNA extraction from swab samples and library preparation. Single-cell genomics is often considered more expensive than metagenomic analysis because of the cost accumulated for each SAG readout. However, the costs per SAG or MAG were 966 yen/SAG (2,320 yen/HQ SAG) for single-cell sequencing and 2,000 yen/MAG (4,571 yen/HQ MAG) for metagenome sequencing. The acquiring efficacies of SAGs or MAGs per sequence depth were 6.3 SAGs/Gb and 1.3 MAGs/Gb. For single-cell genomics, *de novo* genome assembly of one SAG took 5 min and can be done in parallel computation, while metagenomics required 6 h per sample from assembly to output of MAG by binning. It should also be noted that MAG represents species-representative genomes and does not provide strain resolution information. These results indicate that single-cell genomics is an excellent method to obtain strain-resolved genomes in a cost- and data-efficient manner.

One of the limitations of this study is the evaluation of skin bacteria sampling on the recovery of various bacterial species SAGs. We observed the differences in the bacterial species composition between SAG and MAG. The bacterial species that could not be obtained by SAG and could only be obtained by MAG included *Cutibacterium* (*Cutibacterium acnes* 6MAG, *Cutibacterium humerusii* 1MAG) and *Dermacoccus* (*Dermacoccus nishinomiyaensis* 4MAG, *Dermacoccus abyssi* 1MAG). Single-cell genome sequencing can only obtain genomes from intact cells, while metagenomic sequencing can obtain the genome from intact cells and free DNA. In addition, *Cutibacterium acnes* had an average GC% of 60%, and *Dermacoccus nishinomiyaensis* had an average GC% of 69%, suggesting that high GC content may have prevented their acquisition. Therefore, there is room for improvement in terms of (i) sampling the bacteria, (ii) storage until analysis, and (iii) accurate genome amplification from bacteria with higher GC content.

In this study, single-cell genomics recovered high-quality strain-resolved SAGs from the skin microbiome and identified intra-species diversity, especially in *M. osloensis*, characterizing the strain-level MGE profiles, including plasmids and prophages. Even in cohabiting human hosts, skin bacterial strain signatures are unique, which shows microdiversity in each host. The technique obtains massive SAGs, enabling the pangenome analysis of uncultured skin bacteria and considering a potential survival strategy to promote micro diversity in the skin. Genetic and functional differences between bacterial strains are a predictor of *in vivo* competition that stems from the bacterial genome’s need to adapt to nutrient scarcity or its need to safeguard by inhibiting the colonization of other microbes on the skin. Thus, single-cell genomics would help the surveys of the skin microbiome at the strain level, supporting the conventional bacteria isolation and metagenomics approaches by providing massive genome information which reveals microdiverisy in specific skin bacterial strains.

## Data availability statement

Sequencing data have been deposited in the NCBI database under BioProject PRJNA837408 and PRJNA692334.

## Ethics statement

The studies involving human participants were reviewed and approved by School of Science and Engineering at Waseda University. The patients/participants provided their written informed consent to participate in this study.

## Author contributions

KI, TS, KA, TY, and MH conceived and designed the experiments. TS, TY, TE, and AM conducted the genomics experiments and collected the data. KI and KA conducted bioinformatics analyses of single-cell genomic data. KI, TS, and MH analyzed all data and wrote the original manuscript. All authors reviewed and approved the final manuscript.

## Supplementary material

SUPPLEMENTARY FIGURE 1 Comparison of high-quality single-amplified genomes (SAGs) with isolate complete genome and metagenome-assembled genomes (MAGs). Genome size, the number of CDS, and GC% of *M. osloensis* and *S. epidermidis*.

SUPPLEMENTARY TABLE 1 Information of participants for skin bacteria sampling.

SUPPLEMENTARY TABLE 2 Assembly quality of validated single-amplified genomes (SAGs) and metagenome-assembled genomes (MAGs) of skin bacteria.

SUPPLEMENTARY TABLE 3 Assembly quality of high-quality single-amplified genomes (HQ SAGs) of skin bacteria.
