# Supplementary figures and images for: Exploring strain diversity of dominant human skin bacterial species using single-cell genome sequencing
Source: Front Microbiol. 2022 Aug 5;13:955404. doi: 10.3389/fmicb.2022.955404 (PMC9389210; doi:10.3389/fmicb.2022.955404)

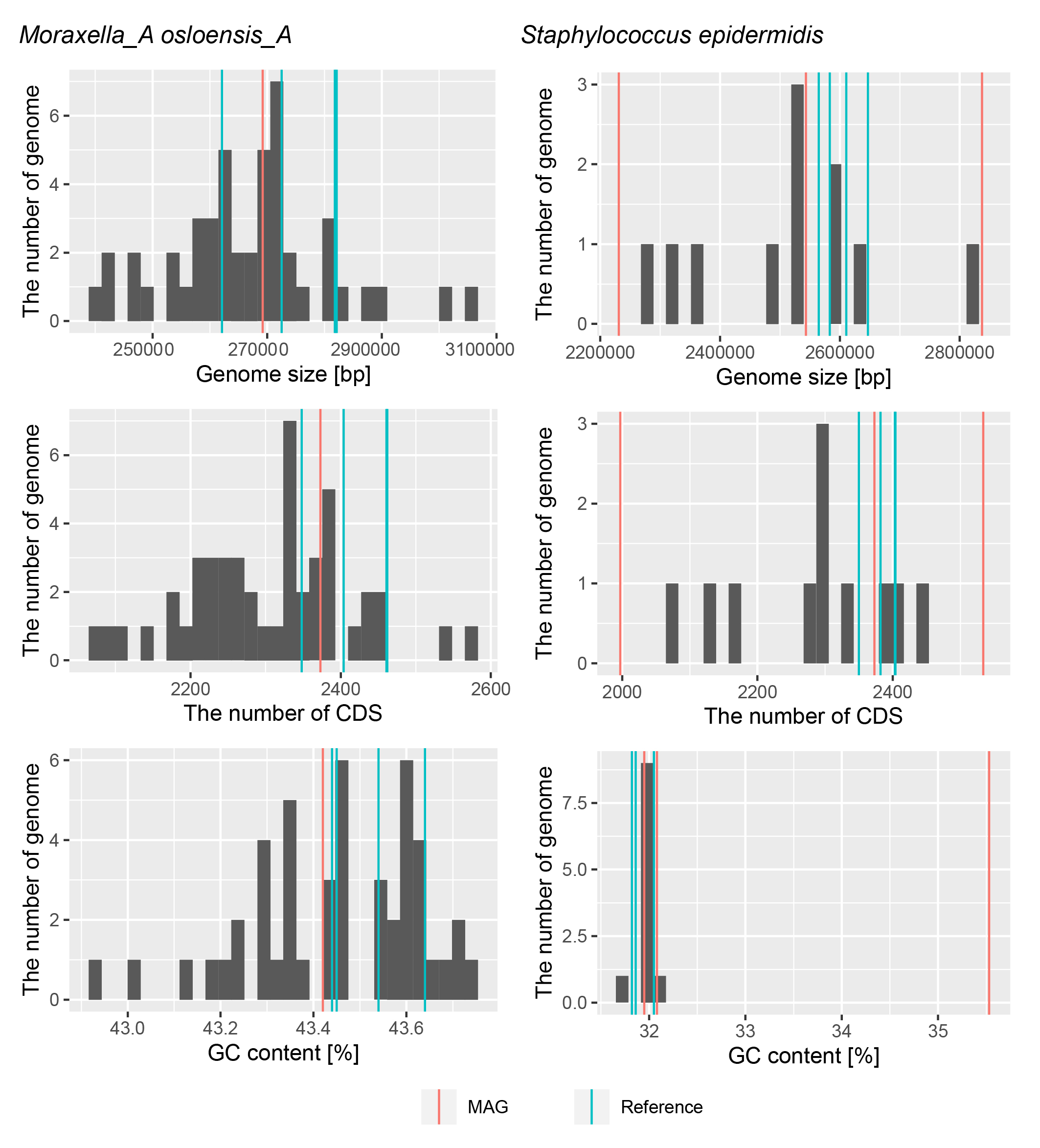

Supplement: SUPPLEMENTARY FIGURE 1 — Comparison of high-quality single-amplified genomes (SAGs) with isolate complete genome and metagenome-assembled genomes (MAGs). Genome size, the number of CDS, and GC% of M. osloensis and S. epidermidis. [file Image_1.JPEG]
